# Supplementary material for: Anti-Allergic and Anti-Inflammatory Effects of Lidocaine-Derived Organic Compounds in a House Dust Mite-Induced Allergic Rhinitis Mouse Model
Source: Biomedicines. 2024 Aug 29;12(9):1965. doi: 10.3390/biomedicines12091965 (PMC11429074; doi:10.3390/biomedicines12091965)
Supplement: Supplementary file 1 [file biomedicines-12-01965-s001.zip › biomedicines-3131317-supplementary.pdf]

Method S1. Docking study to determine interaction between EI341 and IL-4.

The 3D structure of interleukin-4 (IL-4) was obtained from the Protein Data Bank (PDB) using the accession code 1IAR. The protein structure underwent preparation for docking by removing water molecules and heteroatoms via PyMOL. To ensure proper protonation states, hydrogen atoms were added to the protein, and the structure was saved in PDBQT format using AutoDockTools (ADT) [1,2]. The novel compound EI341 was designed, with its 3D structure generated using ChemSketch. The structure was optimized with the MMFF94 force field in Avogadro, and the resulting geometry was saved in PDB format. Using ADT, the ligand was converted to PDBQT format, ensuring proper definition of rotatable bonds.

Molecular docking simulations were conducted using AutoDock Vina 1.1.2. A grid box encompassing the entire enzyme was defined to explore all potential binding sites. The grid box dimensions were set to cover the entire receptor, with the center coordinates and box size specified as follows: Center coordinates: (20, 20, -12), Box size: (30, 30, 30). Default docking parameters were used, with an exhaustiveness of 8 to balance computational efficiency and accuracy.

AutoDock Vina was executed to predict the binding affinity and pose of EI341 within the active site of IL-4. Ten top binding poses were generated, ranked by their binding affinities (in kcal/mol). The pose with the lowest binding energy was selected for further analysis.

Docked poses were visualized and analyzed using PyMOL (The PyMOL Molecular Graphics System, Version 1.3, Schrödinger, LLC.) and LigPlot+ to identify key interactions between EI341 and IL-4 [3]. Hydrogen bonds, hydrophobic interactions, and other significant contacts were documented to understand the binding mechanism of EI341.

References.

21. Trott, O.; Olson, A.J. AutoDock Vina: improving the speed and accuracy of docking with a new scoring function, efficient optimization, and multithreading. *J Comput Chem* **2010**, *31*, 455-461, doi:10.1002/jcc.21334.
22. Eberhardt, J.; Santos-Martins, D.; Tillack, A.F.; Forli, S. AutoDock Vina 1.2.0: New Docking Methods, Expanded Force Field, and Python Bindings. *J Chem Inf Model* **2021**, *61*, 3891-3898, doi:10.1021/acs.jcim.1c00203.
23. Laskowski, R.A.; Swindells, M.B. LigPlot+: multiple ligand-protein interaction diagrams for drug discovery. *J Chem Inf Model* **2011**, *51*, 2778-2786, doi:10.1021/ci200227u.

Result S1. The interaction between EI341 and interleukin (IL)-4 and its receptor.
To evaluate the impact of the novel compounds on the interaction between IL-4 and its receptor, a comprehensive docking study was performed, focusing on compound EI341 as a representative example. The docking results indicated that EI341 exhibits a moderate binding affinity to its target, with a binding energy of -6.193 kcal/mol.

As illustrated in Figure S3F, which displays both 2D and 3D representations of the docking interactions, EI341 is strategically positioned within the groove between IL-4 and its receptor. This placement suggests that EI341 may influence the protein-protein interaction. Detailed analysis of the binding interactions revealed that EI341 forms a hydrogen bond with Asn130. Additionally, several hydrophobic interactions were observed between EI341 and the residues Lys12, Asp125, Tyr127, Tyr129, and Pro124.

These findings imply that EI341 has the potential to modulate the IL-4/receptor interaction, which could be significant for its biological activity and therapeutic potential.

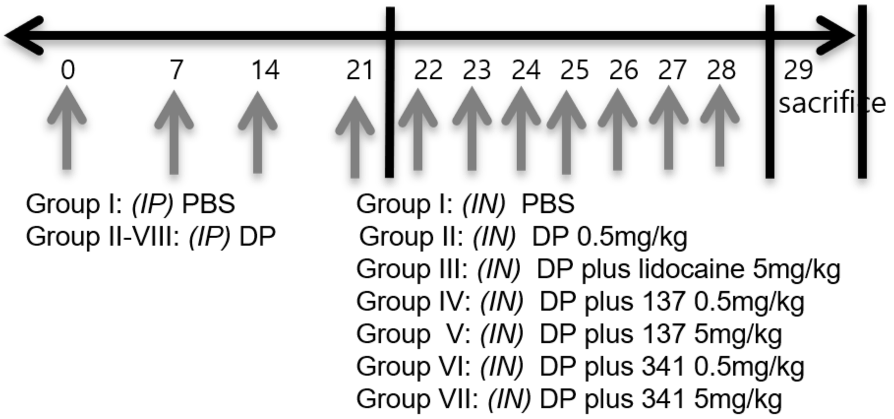

Figure S1. Schematic diagram of *Dermatophagoides pteronyssinus* (DP)-induced allergic rhinitis mouse model. DP was intraperitoneally injected on day 0, 7, 14, and 21 then stimulated with DP from day 22 to 28. Mice were pretreated with lidocaine (5 mg/kg) or lidocaine analogs EI137 and EI341 (0.5 and 5 mg/kg) in each experimental group. IP, intraperitoneal; PBS, phosphate buffered saline; gray arrow, intranasal application.

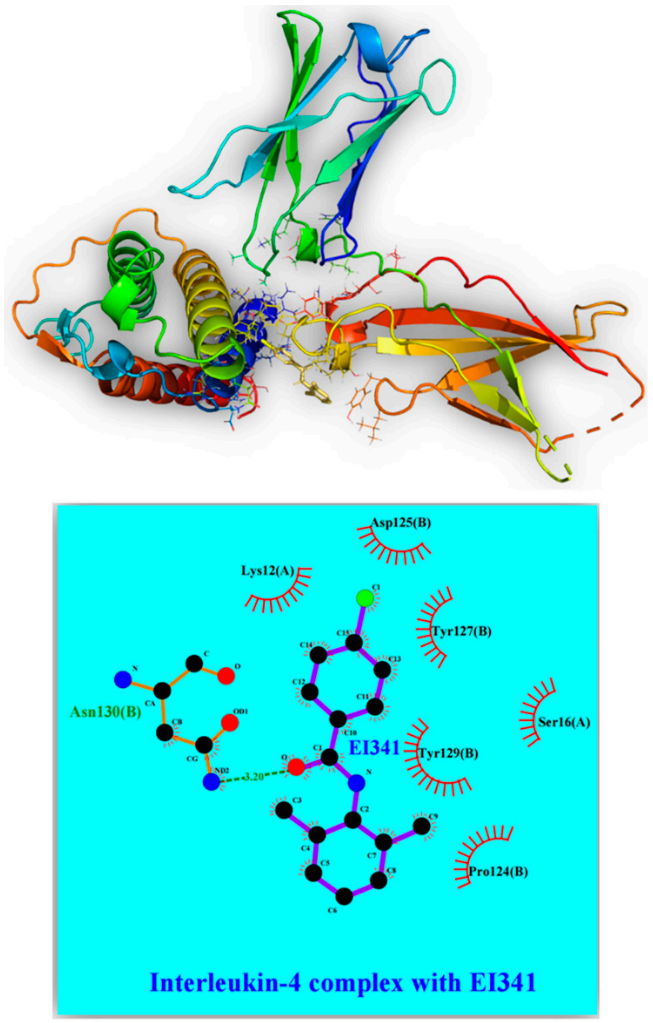

Asn, Asparagine; Lys, Lysine; Asp, Aspartic acid; Tyr, Tyrosine; Pro, Proline.

Figure S2. 3D and 2D representation of interaction between EI341 and interleukin-4.
